# Electrospun PLA-Based Biomaterials Loaded with *Melissa officinalis* Extract with Strong Antioxidant Activity

**DOI:** 10.3390/polym15051070

**Published:** 2023-02-21

**Authors:** Nikoleta Stoyanova, Mariya Spasova, Nevena Manolova, Iliya Rashkov, Mariana Kamenova-Nacheva, Plamena Staleva, Maya Tavlinova-Kirilova

**Affiliations:** 1 Laboratory of Bioactive Polymers, Institute of Polymers, Bulgarian Academy of Sciences, Acad. G. Bonchev St, bl. 103A, BG-1113 Sofia, Bulgaria; 2 Laboratory Organic Synthesis and Stereochemistry, Institute of Organic Chemistry with Centre of Phytochemistry, Bulgarian Academy of Sciences, Acad. G. Bonchev St, bl. 9, BG-1113 Sofia, Bulgaria; 3 Laboratory for Extraction of Natural Products and Synthesis of Bioactive Compounds, Research and Development and Innovation Consortium, Sofia Tech Park JSC, 111 Tsarigradsko Shose blvd., BG-1784 Sofia, Bulgaria

**Keywords:** *Melissa officinalis*, electrospinning, PLA, PEG, antioxidant activity

## Abstract

In the present study, the plant extract *Melissa officinalis* (*M. officinalis)* was successfully loaded in polymer fibrous materials on the basis of a biodegradable polyester–poly(L-lactide) (PLA) and biocompatible polyether–polyethylene glycol (PEG) by applying the electrospinning method. The optimal process conditions for the preparation of hybrid fibrous materials were found. The extract concentration was varied—0, 5 or 10 wt% in respect of the polymer weight, in order to study its influence on the morphology and the physico-chemical properties of the obtained electrospun materials. All the prepared fibrous mats were composed of defect-free fibers. The mean fiber diameters of the PLA, PLA/*M. officinalis* (5 wt%) and PLA/*M. officinalis* (10 wt%) were 1370 ± 220 nm, 1398 ± 233 nm and 1506 ± 242 nm, respectively. The incorporation of the *M. officinalis* into the fibers resulted in slight increase of the fiber diameters and in increase of the water contact angle values to 133°. The presence of the polyether in the fabricated fibrous material assisted the wetting of the materials imparting them with hydrophilicity (the value of the water contact angle become 0°). Extract-containing fibrous materials displayed strong antioxidant activity as determined by the 2,2-diphenyl-1-picryl-hydrazyl-hydrate free radical method. The DPPH solution color changed to yellow and the absorbance of the DPPH radical dropped by 88.7% and 91% after being in contact with PLA/*M. officinalis* and PLA/PEG/*M. officinalis* mats, respectively. These features revealed the *M. officinalis*—containing fibrous biomaterials promising candidates for pharmaceutical, cosmetic and biomedical use.

## 1. Introduction

Since ancient times, humans have known about plants as a medicinal cure. However, in recent years, plant extracts have attracted increased interest due to their natural origin and complex of desirable properties [1]. Plant extracts have high content of various bioactive compounds such as polyphenols [2] and carotenoids [3] and, therefore, could be used as therapeutic drugs [4], health foods [5], cosmetics [6], chemical alternatives [7], biopesticides, etc. [8].

*Melissa Officinalis* L. (*lemon balm* or) is a cultivated perennial lemon-scented herb of the Lamiaceae family [9]. This medical plant commonly grows in south-central Europe, North Africa, the Mediterranean region, and Central Asia [10]. Studies have shown that *Melissa officinalis* contains mainly alkaloids, tannins, flavonoids, saponins, and phenolic compounds [11]. The main active constituents of this medical plant are volatile compounds such as geranial, neral, citronellal and geraniol; triterpenes-ursolic acid and oleanolic acid; phenolic compounds such as rosmarinic acid (RA), caffeic acid, and protocatechuic acid and flavonoids-quercetin, rhamnocitrin, luteolin [12].

Many pharmacological studies reported the diverse favorable effects of *Melissa officinalis.* This medical plant possesses antioxidant [13,14], cytostatic [15,16] and anti-inflammatory effects [17]. In recent decades, several studies revealed the anxiolytic effects of this plant. The methanol extract of *M. officinalis* and its main component rosmarinic acid (RA) showed GABA-T inhibitory activity on rat brain [18]. Oral administration of the hydroalcoholic and ethanolic extracts of the plant induced anxiolytic-like effects [19]. The use of *M. officinalis* in the treatment of depression [20], dementia, amnesia [21] and diabetes [22] is described as well.

In recent years, the development of, and advances in, nanotechnology allow the creation of novel materials with improved properties for diverse applications [23]. Electrospinning is a modern, versatile and cost-effective method that enables fabrication of continuous nano- and micro-fibrous materials with adjustable structure, properties, and functions [24]. Electrospun fibers possess very large surface area to volume ratios, high porosity, good mechanical properties, flexibility in functionalization, etc. [25]. Moreover, nanofibrous mats can be modified in order to meet the needs of certain applications, such as by incorporation of functional additives or active compounds into the spinning solution [26,27], formation of a coating on their surface [28], or interfacial interaction or polymerization [29,30]. These modification approaches are leading to preparation of electrospun nanofibers with advantages for applications in different fields such as medicine (drug delivery, tissue engineering, wound healing, enzyme immobilization), cosmetics, the food industry, agriculture, water and air filtration, energy, biotechnology, and sensors [31,32,33].

Recently, many research studies have proved that eco-friendly materials for medicinal purposes can be created by combining plant extracts with natural or synthetic polymers. Up until now, diverse plant extracts have been loaded into electrospun fibers such as: *Coptis chinensis* [34] and *Tridax procumbentsis* [35] in poly(vinyl alcohol) nanofibers, *Inula graveolens* (L.) in polycaprolactone (PCL) [36], *eucalyptus citriodora* in zein [37], *Curcuma longa* in cellulose acetate [38], *Portulaca oleracea* in PLA [39], etc.

Polylactide (PLA) is a biodegradable thermoplastic polymer derived from renewable, organic sources such as corn starch or sugar cane [40]. The advantages of PLA compared to other biopolymers are numerous, including: it is eco-friendly, biodegradable, recyclable, compostable and non-toxic. PLA hydrolyzes to its constituent α-hydroxy acid when implanted in the human body or in other living organisms. Then, it is incorporated into the tricarboxylic acid cycle and excreted. Moreover, PLA possesses better thermal processability compared to other biopolymers such as polyhydroxyalkanoates (PHA), and poly(ε-caprolactone) (PCL). However, PLA has some drawbacks such as: poor toughness, lack of reactive side-chain groups, slow degradation rate and relatively high hydrophobicity that can lead to low cell affinity and some inflammatory response from the living host [41].

To our knowledge, there is no study in the literature which has reported on the incorporation of a *M. officinalis* plant extract into electrospun polymer fibers and studies their properties.

In the present work, for the first time, novel biomaterials loaded with *M. officinalis* plant extract were fabricated by electrospinning. Optimal process parameters were determined in order to obtain uniform fibers. In order to impart hydrophilicity that could assist the action of the extract, a second polymer which was water soluble was added to the polymer matrix. The effect of the incorporation of the plant extract and PEG to the PLA fibers and their properties were studied. Additionally, in the view of the possible materials application in biomedical field, the antioxidant activity of all fibrous materials was investigated.

## 2. Materials and Methods

### 2.1. Used Materials

Poly(L-lactide) (PLA, Ingeo™ Biopolymer 4032D, NatureWorks LLC—USA; Minnetonka, MN, USA; M_W_ = 259,000 g/mol; M_W_/M_n_ = 1.94; as determined by size-exclusion chromatography using polystyrene standards), polyethylene glycol (PEG 100,000, Serva, Heidelberg, Germany) were used. Dichloromethane (DCM, Merck, Darmstadt, Germany) and ethanol (abs. EtOH, Merck, Darmstadt, Germany) of analytical-grade purity were used. The 2,2-Diphenyl-1-picrylhydrazyl (DPPH) was supplied from Sigma-Aldrich (Darmstadt, Germany). All chemicals used were of analytical grade and were used as received without any further purification.

Plant material from cultivated Lemon balm (*Melissa officinalis*) was provided by the company “Essential Oils and Herbs“ Ltd. (grown in the village of Blatets, Bulgaria).

The plant extract was prepared by stirring 268 g of air-dried and ground leaves, flowers and stems of lemon balm (*M. officinalis*) in 70% of aqueous methanol (solid/liquid ratio of 1/30 (g/mL) for 24 h at room temperature. Further, the mixture was filtrated and the methanol was evaporated under reduced pressure using a rotary evaporator. The aqueous residue was spray dried on a Buchi Mini Spray Dryer B-290 and 23.01 g (8.6% yield) of dry extract of *M. officinalis* was isolated as a yellow-green powder.

### 2.2. Instrumentation and Chromatographic Conditions for Chemical Characterization of Dry Extract of M. officinalis

The chemical characterization of dry extract of *M. officinalis* was performed by HPLC-DAD-ESI/MS on a Shimadzu LC-2040C 3D Nexera-i and Shimadzu LCMS 2020 (single quadrupole). Separation of compounds was carried out on a column Force C18 (Restek, Bellefonte, PA, USA), 3 μm, 150 mm × 4.6 mm, thermostated at 40 °C. The UV spectra were recorded from 190 to 800 nm. The ion spray voltage was set in the negative mode at −4.50 kV; scan range: 100–1000 *m/z*; interface temperature: 350 °C; desolvation line: 250 °C; heat block: 200 °C; nebulizing gas flow: 1.5 L/min and drying gas flow: 15 L/min. The solvents used were: (A) 0.1% formic acid in water and (B) acetonitrile. The following gradient program was performed: 12% B isocratic for 5 min, 12–30% B over 45 min, 30–90% B over 5 min, 90% B isocratic for 1 min, 90–12% B over 1 min, and re-equilibration of the column for 5 min. The flow rate was 0.5 mL/min and the injected volume was 2 μL. The extract was dissolved in methanol at a concentration of 550 mg/L. At the same conditions quantification of rosmarinic acid was performed in the extract. The wavelength selected for the quantification was 330 nm. Identification was accomplished by comparing the retention times (Rt) and UV spectra of the corresponding peak in the sample to those of the standard. The amount of rosmarinic acid was calculated utilizing a calibration curve (1–50 mg/L, r^2^ = 0.9999).

### 2.3. Fabrication of Fibrous Mats by Electrospinning

Different types of fibrous materials, including PLA, PLA/PEG, PLA/*M. officinalis* and PLA/PEG/*M. officinalis* were fabricated by electrospinning. Prior to electrospinning, the following spinning solutions were prepared in a mixture of dichloromethane/ethanol 80/20 *v*/*v*: (1) PLA (10 wt%), (2) PLA/PEG (80/20 *w*/*w*), (3) PLA/*M. officinalis* (5 and 10 wt%) and (4) PLA/PEG/*M. officinalis* (5 and 10 wt%). The total polymer concentration was 10 wt%.

The prepared solutions were then transferred into a 5 mL syringe equipped with a metal needle (size: 20GX1½″) whose tip was attached to the positively charged electrode. The electrode was connected to a specially constructed high voltage power source that could generate positive DC voltages between 10 and 30 kV. The electrospun fibers were collected on a grounded rotating drum, which had a diameter of 45 mm. The collector was placed 15 cm away from the needle’s tip. The collector rotation speed was 1000 rpm. The spinning solutions were delivered by an infusion pump (NE-300 Just InfusionTM Syringe Pump, New Era Pump Systems Inc., Farmingdale, NY, USA) at a constant feed rate of 3 mL/h. The other parameters were as follows: applied voltage −25 kV, room temperature −21 °C and a relative humidity of 53%. All the prepared fibrous materials were placed under reduced pressure at 25 °C to remove any remaining solvent.

### 2.4. Complex Characterization of the Fibrous Materials

The dynamic viscosity of the prepared spinning solutions were determined on a Brookfield DV-II+ Pro programmable viscometer equipped with a sample thermostatic cup and a cone spindle for the one/plate option operating at room temperature −25 °C.

Scanning electron microscopy was used to study in detail the morphology of the fabricated electrospun fibrous materials. Prior to SEM observation on Jeol JSM-5510 (JEOL Co., Ltd., Tokyo, Japan), the materials were vacuum-coated with gold on a Jeol JFC-1200 fine coater for 60 s. The captured SEM micrographs were used to determine the mean fiber diameter and the standard deviation by using Image J software and measuring at least 30 fibers from SEM images [42].

Attenuated total reflection Fourier transform infrared (ATR-FTIR) spectroscopic analysis was performed on IRAffinity-1 spectrophotometer (Shimadzu, Kyoto, Japan) equipped with a MIRacle ATR accessory (diamond crystal, depth of penetration of the IR beam into the material is 2 μm). The spectra were recorded in range 4000 to 500 cm^−1^ with a spectral resolution of 4 cm^−1^ using a DLATGS detector connected with a temperature controller. H_2_O and CO_2_ content of all spectra was adjusted with IRsolution’s software. All the samples were dried under reduced pressure before analysis.

The hydrophobic/hydrophilic balance of the surface of the prepared fibrous materials was studied by measuring the static contact angle on a DSA 10-MK2 drop shape analyzer system (Krüss, Hamburg, Germany) at 20 ± 0.2 °C. Contact angles of the fibrous materials were measured by dropping a deionized water droplet with volume of 10 μL controlled by a computer dosing system. The droplet’s temporal photographs were captured. Computer analysis of the obtained images was used to measure the water contact angles. The represented mean contact angle value is a result of 20 measurements taken on various regions of the mat surfaces.

X-ray diffraction analysis (XRD) was performed to analyze the crystalline structure of the plant extract and fabricated electrospun fibrous materials. D8 Bruker Advance powder diffractometer (Bruker, Billerica, MA, USA) equipped with a filtered CuK radiation source and a luminous detector (step of 0.02° and counting time of 1 s/step) was used to record the XRD patterns.

Mechanical properties of the fibrous materials were determined by tensile measurements performed on a single column system for mechanical testing, INSTRON 3344, equipped with a loading cell of 50 N and Bluehill universal software. The initial length between the clamps was 40 mm and the used stretching rate was 10 mm/min. The fibrous samples were cut with dimensions of 20 × 60 mm^2^. A Digital Thickness Gauge FD 50 (Kafer GmbH, München, Germany) was used to determine the thickness of the fibrous materials. The average thickness was ca. 250 μm ± 20 nm.

Thermogravimetric analysis (TGA) was performed on Perkin Elmer TGA 4000 (Waltham, MA, USA) at 10 °C/min heating rate under argon flow of 60 mL/min. Pyris v.11.0.0.0449 software was used for instrument control, data collecting, and data processing.

A radical scavenging assay with 2,2-diphenyl-1-picrylhydrazyl (DPPH) was used to determine the antioxidant activity of the *M. officinalis* extract and the antioxidant capacity of the fabricated fibrous materials. Ethanol solution of the plant extract and PLA, PLA/*M. officinalis* (10 wt%), PLA/PEG and PLA/PEG/*M. officinalis* (10 wt%) electrospun samples with weight of 0.5 mg were immersed in 3 mL of ethanol DPPH solution (1 × 10^−4^ M). Then, the solutions were placed for 30 min at 20 °C in the dark. A DU 800 UV-Vis spectrophotometer (Beckman Coulter, Brea, CA, USA) was used to characterize the solutions’ absorbance at 517 nm in order to determine how many DPPH radicals were still present in the solution. The antioxidant activity (AA%) was calculated by using the following equation:

$$\mathrm{Inhibition},\;\mathrm{AA},\%=\left[\frac{(A_{\mathrm{DPPH}}-A_{\mathrm{sample}})}{A_{\mathrm{DPPH}}}\right]\times \;100\;$$

In the used equation, the A_sample_- A_DPPH•_ is the solution absorption at 517 nm after the addition of the extract solution or fibrous materials. A_DPPH•_ presents the absorption for DPPH• solution at 517 nm. Each experiment was performed three times.

### 2.5. Statistical Analysis

The results’ data were displayed as means ± standard deviation (SD). One-way analysis of variance (ANOVA) and the post hoc comparison test (Bonferroni) were used with the GraphPAD PRISM program, version 5, to evaluate the statistical significance of the data (GraphPad Software Inc., San Diego, CA, USA). Statistics were considered to be significant for values of * *p* < 0.05, ** *p* < 0.01 and *** *p* < 0.001.

## 3. Results and Discussion

### 3.1. Identification of Main Phenolic Compounds and Quantification of Rosmarinic Acid in the Dry Extract of M. officinalis

**Figure 1:**
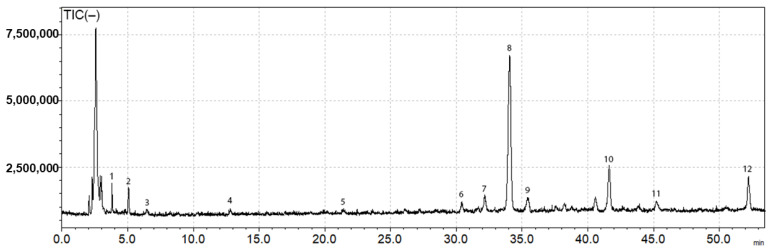
Total ion chromatogram (TIC) of *M. officinalis* extract in negative ionization mode.

The identification of the main phenolic compounds in the extracts of *M. officinalis* was performed by HPLC-DAD-ESI/MS. ESI negative mode was performed since it is more sensitive for phenolic compounds. Peak identification was carried out by comparing retention times, UV and mass spectra of individual components with those of standards and literature data. Total ion chromatogram (TIC) in negative mode of *M. officinalis* extract is shown on the Figure 1 and the data of the tentatively identified compounds is presented in Table 1.

The LC-MS data showed the presence of rosmarinic acid—a caffeic acid ester (Figure 1; peak 8, rt: 34.1 min, [M-H]^−^: 359) and caffeic acid (Figure 1; peak 4, rt: 12.8 min, [M-H]^−^: 179). Their identity was confirmed by comparison with commercial standards. Other compounds identified included RA derivatives such as sulphated rosmarinic acid (Figure 1; peak 7, rt: 32.2 min, [M-H]^−^: 439), sagerinic acid—a rosmarinic acid dimer (Figure 1, peak 6, rt: 30.4 min [M-H]^−^: 719), lithospermic acid A (Figure 1, peak 10, rt: 41.6 min M–H: 537), salvianolic acid isomer and salvianolic acid C derivative (Figure 1, peak 11 and 12, rt: 45.2 and 52.2 min, [M-H]^−^: 717 and 715, respectively). Other phenolic acid identified were 3-(3,4-dihydroxyphenyl)-lactic acid (Figure 1; peak 2, rt: 5.1 min, [M-H]^−^: 197; dimer [2M-H]^−^: 395) and caftaric acid (Figure 1; peak 3, rt: 6.5 min, [M-H]^−^: 311). The cyclic polyol quinic acid (Figure 1; peak 1, rt: 3.0 min, [M-H]^−^: 191) was also detected in the sample. Finally, peak 9 was identified as luteolin 7-O-glucuronide, the only flavonoid found in the extract (Figure 1, rt: 35.5 min, [M-H]^−^: 461). Its identification was based on the fragmentation found in the literature (Table 1). Since, rosmarinic acid was identified as a major component in the extract, quantification studies for this compound were performed. The amount of rosmarinic acid found in the extract was 76.27 ± 0.1 mg/g. Numerous studies have shown a direct relationship between the presence of RA and the in vitro bioactivities demonstrated by extracts [12].

### 3.2. Morphological Analysis of the Obtained Electrospun Materials

**Figure 2:**
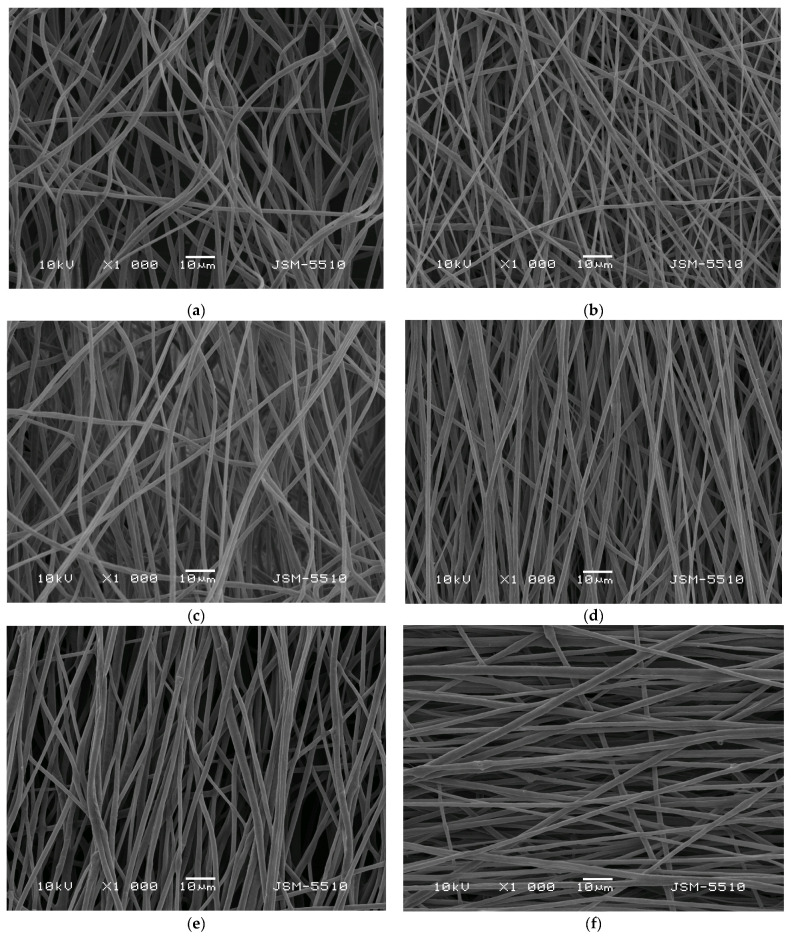
SEM images of fibrous mats based on: (**a**) PLA, (**b**) PLA/PEG, (**c**) PLA/*M. officinalis* (5 wt%)*; (***d**) PLA/PEG/*M. officinalis* (5 wt%)*,* (**e**) PLA/*M. officinalis* (10 wt%) and (**f**) *PLA*/PEG/*M. officinalis* (10 wt%).

The morphology of the obtained electrospun fibers was assessed by using scanning electron microscopy (SEM). Figure 2 reveals the morphology of the prepared fibrous materials based on: (a) PLA, (b) PLA/PEG, (c) PLA/*M. officinalis* (5 wt%); (d) PLA/PEG/*M. officinalis* (5 wt%), (e) PLA/*M. officinalis* (10 wt%) and PLA/PEG/*M. officinalis* (10 wt%). All the materials were obtained by one-pot electrospinning. As seen in Figure 2, the all fibrous mats were composed of cylindrical and defect-free fibers. The incorporation of PEG and the plant extract to the PLA solution and subsequent electrospinning do not provoke the appearance of process instability and defect formation. The average fiber diameter of the prepared fibers was determined by using the acquired SEM images. The measured mean fiber diameters of the PLA, PLA/*M. officinalis* (5 wt%) and PLA/*M. officinalis* (10 wt%) were 1370 ± 220 nm, 1398 ± 233 nm and 1506 ± 242 nm, respectively. It was ascertained that the loading of the plant extract of *M. officinalis* resulted in slight increase of the measured fiber diameters, while preserving their cylindrical form. This slight increase in fiber diameters and their distributions is most probably due to increase of the dynamic viscosity of the spinning solutions. The measured values of the PLA, PLA/*M. officinalis* (5 wt%) and PLA/*M. officinalis* (10 wt%) spinning solutions were 1700 cP, 1767 cP and 1802 cP, respectively. Furthermore, the incorporation of a second polymer–PEG to the spinning solution had influence on the viscosity and mean fiber diameters as well. The PLA/PEG, PLA/PEG/*M. officinalis* (5 wt%) and PLA/PEG/*M. officinalis* (10 wt%) spinning solutions have the following dynamic viscosity values: 1245 cP, 1500 cP and 1528 cP. The incorporation of PEG to PLA solution resulted in decrease of the viscosity and fiber’ diameters compared to the PLA one. The loading of *M. officinalis* extract to the PLA or PLA/PEG resulted in a slight raise in solution viscosity and in the resulted fiber diameters. Increasing the concentration of the crude plant extract in the spinning solutions and, consequently, in the fibrous mats led to preparation of thicker fibers.

### 3.3. ATR-FTIR Analysis

**Figure 3:**
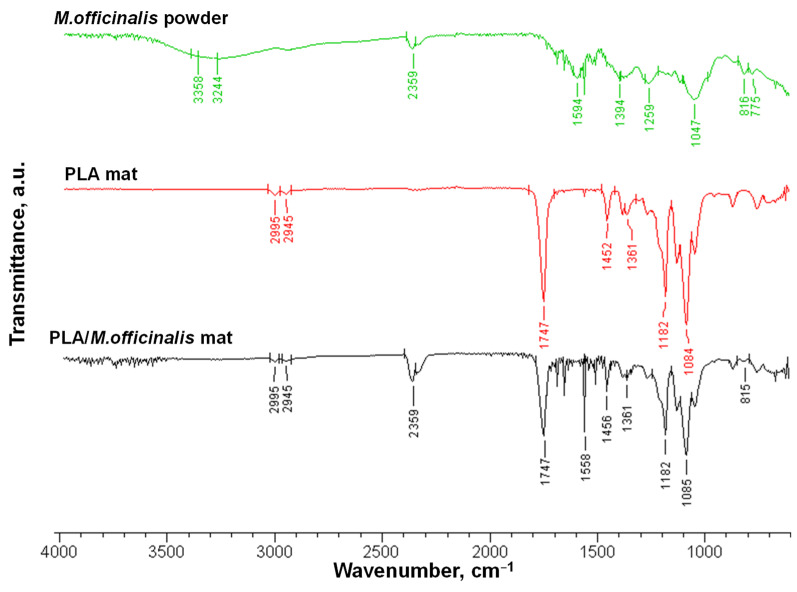
ATR-FTIR spectrum of *M. officinalis* (powder), PLA fibrous mat and PLA/*M. officinalis* fibrous mat.

PLA, PLA/PEG, PLA/*M. officinalis* and *PLA*/PEG/*M. officinalis *fibrous materials**, as well as *M. officinalis* extract (powder) were characterized by ATR-FTIR spectroscopy. The recorded spectra of the extract, PLA and PLA/*M. officinalis* were presented in Figure 3. The methanolic extract of *M. officinalis* is a complex mixture which could be easily seen from its FTIR spectrum where a number of absorption peaks are present. The strong bands at 3358 and 3244 cm^−1^ could be assigned to O-H stretch, H-bonded corresponded to alcohols and phenols, 2359 cm^−1^ assigned for single aldehyde, 1594 cm^−1^ indicates the fingerprint region of C-O stretching. The absorption bands at 1259 cm^−1^ and 1394 cm^−1^ were attributed to the symmetric deformation of the –CH_3_ group. FTIR spectra of *M. officinalis* extract displays a band corresponding to C-O stretching vibrations (1047 cm^−1^). The absorption bands at 816 cm^−1^, as well as at 775 cm^−1^, are due to bending of the aromatic ring of the polyphenols [47]. In the FTIR spectra of the PLA mat, characteristic stretching frequencies for C=O, –CH_3_ asymmetric, –CH_3_ symmetric, and C–O, at 1747, 2995, 2945 and 1084 cm^−1^ were presented [39]. It was determined that the bending frequencies for –CH_3_ asymmetric and –CH_3_ symmetric are identified at 1452 and 1361 cm^−1^, respectively. In the spectrum of the PLA/*M. officinalis* fibrous mat, characteristic bands for PLA as well as for the lemon balm (1748 and 2359 cm^−1^, respectively) were detected. The presence of PEG in PLA/PEG and PLA/PEG/*M. oficinalis* mats resulted in detecting additional bands at 2881, 2332, 1359 and 963 cm^−1^, characteristic for PEG (Supplementary material Figure S1). In the IR spectrum of PLA/PEG/*M. officinalis*, fibrous material bands characteristic for PLA, PEG and plant extract are presented, proving the successful incorporation of the *M. officinalis* in the PLA/PEG polymer matrix.

### 3.4. Water Contact Angle

One of the key characteristics of materials is their hydrophilic/hydrophobic properties [48]. It is known that the wetting behavior depends on the chemical nature of the solid and liquid phases. Hydrophilic surfaces show strong affinity to water and the water droplet is spreading rapidly on this kind of surfaces. The degree of hydrophilicity/hydrophobicity of the materials’ surface could be determined by measuring the contact angle between the liquid and solid phases.

**Figure 4:**
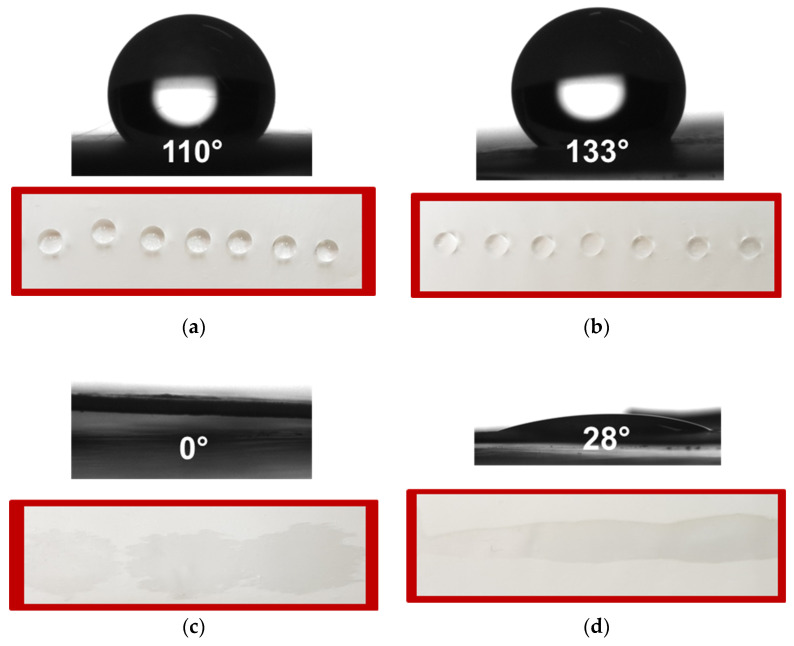
Digital images of distilled water drops (10 μL) placed on surfaces of fibrous mats based on: (**a**) PLA, (**b**) PLA/*M. officinalis* (10 wt%), (**c**) PLA/PEG and (**d**) PLA/PEG/*M. officinalis* (10 wt%).

Therefore, the water contact angles values and the shape of the water drop on the prepared fibrous mats were detected. Digital images of the water droplets that were deposited on the fiber mats’ surfaces were displayed in Figure 4. The value of the contact angle of PLA fibers is 110° ± 2.6°. This value is in very good agreement with the values found in the literature, proving that the PLA fibrous mat is hydrophobic [49]. The incorporation of the *M. officinalis* crude extract resulted in slight increase of the measured water contact angle. The determined values for the PLA mat loaded with 5 wt% and 10 wt% of *M. officinalis* were 121° ± 2.7° and 133° ± 2.5°, respectively. The incorporation into the fibrous matrix of a second polymer–PEG which is water-soluble resulted in hyrophilization of the prepared hybrid mats. The water contact angle values right after dropping the water droplets on the PLA/PEG and *PLA/*PEG/*M. officinalis* (10 wt%) mats were 0° and 28°. After 2 min, the water drops completely absorb into the fibrous materials proving that the PEG-containing fibers are hydrophilic with water contact angle of 0°. This imparted hydrophilicity is a considered as crucial characteristic for attaining a rapid therapeutic action of the biologically active compounds from the extract when applying the obtained materials in medical and pharmaceutical fields.

### 3.5. X-ray Diffraction Analysis

**Figure 5:**
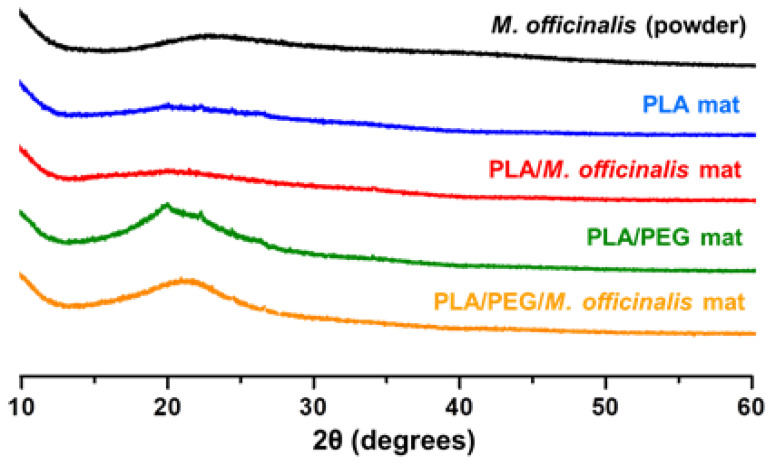
XRD patterns of *M. officinalis* extract (powder), PLA mat, PLA/*M. officinalis* (10 wt%) mat, PLA/PEG mat and PLA/PEG/*M. officinalis* (10 wt%) mat.

Figure 5 presents the XRD patterns of *M. officinalis* powder extract and mats composed of PLA, PLA/*M. officinalis* (10 wt%), PLA/PEG and PLA/PEG/*M. officinalis* (10 wt%) which were recorded in the range 2θ 10–60°. As seen from the XRD pattern, *M. officinalis* powder diffractogram showed no crystalline planes. This finding is similar to the results reported by Santos et al., showing no crystallinity in the *C*. *officinalis* extract [50]. As expected, in the XRD spectra of electrospun PLA and PLA/*M. officinalis* fibrous mats, no diffraction peaks are detected, revealing that materials on the basis of PLA are amorphous. In the XRD pattern of PLA/PEG fibers, one of the PEG well-distinguished peaks appeared at 19.3° assigned to a set of planes (120) [51]. However, the X-ray diffraction studies revealed that the *M. officinalis* plant extract and the obtained electrospun mats were amorphous. Except the peak for the PEG crystallinity, in all patterns the existence of an amorphous halo is presented. This means that biologically active substances in the crude extract as well as in the electrospun fibrous mats are in an amorphous form.

### 3.6. Mechanical Properties

**Figure 6:**
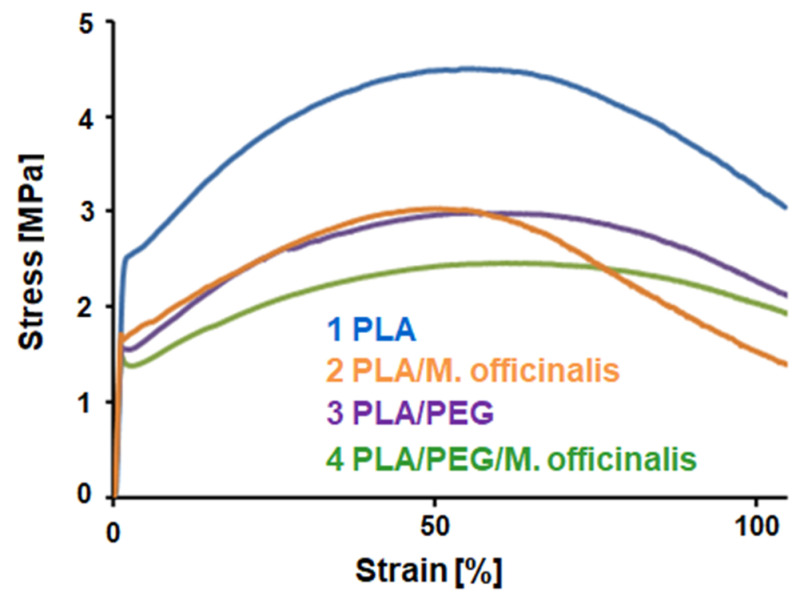
Stress–strain curves of fibrous PLA, PLA/*M. officinalis* (10 wt%), PLA/PEG and PLA/PEG/*M. officinalis* (10 wt%) mats obtained by electrospinning.

One of the most important properties of the electrospun fibers is their mechanical characteristics, which plays a key role in determining the fibers’ applications. The mechanical characteristics of the fibrous mats depend strongly on measurement technique, conditions of fiber fabrication, fiber orientation, point bonding, crosslinking, etc. Moreover, additional component(s) to the spinning solution might have significant effect on the mechanical behavior of the resulting hybrid fibers. Therefore, it is crucial to study the influence of the extract on the mechanical properties of the hybrid PLA/*M. officinalis* mats and PLA/PEG/*M. officinalis*. Mechanical characteristics of the obtained electrospun mats were determined using a single-column tensile testing machine. The typical stress–strain curves of PLA, PLA/*M. officinalis*, PLA/PEG and PLA/PEG/*M. officinalis* mats are presented in Figure 6. The highest value of the tensile strength was determined for the PLA fibrous mat −4.6 MPa. The incorporation of the *M. officinalis* extract resulted in a decrease of the tensile strength to 3 MPa. The decrease in mechanical characteristics might be due to the incorporation of low molecular weight compound in the PLA matrix, which might have generated weak spots when the tensile test was carried out. The incorporation of PEG affected the mechanical properties as well. The detected decease could be attributed to the molecular weight of the used PEG. Despite the slight decrease in the mechanical characteristics of the hybrid fibers, the fibrous materials preserve good mechanical properties.

### 3.7. Thermal Analysis

**Figure 7:**
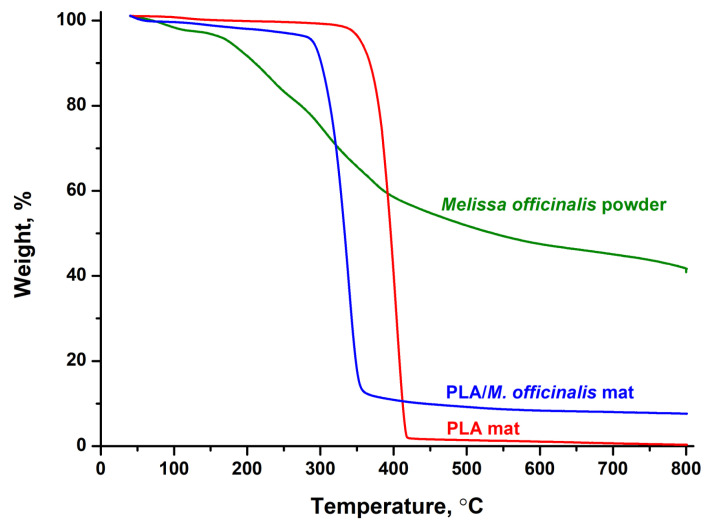
TG thermograms of: *Melissa officinalis* powder, PLA mat and PLA/*M. officinalis* mat in the range 50 to 800 °C.

The thermal behavior of the plant extract (powder) and of the obtained novel fibrous materials of PLA and PLA/*M. officinalis* was determined by TGA analysis. The temperature range was from 50 to 800 °C. The TG thermograms of *M. officinalis* extract, PLA fibers and PLA/*M. officinalis* fibers were shown in Figure 7. As seen from Figure 7, the *M. officinalis* extract showed degradation in four steps. The first weight loss (~3.5%) taking place between 50 °C to 110 °C is attributed to the loss of adsorbed water. The second weight loss (110 °C to 250 °C) of about 16.5%, third weight loss between 250 °C and 350 °C of ∼16% and fourth loss of about ∼23% are attributed to the decomposition of phenolic compounds and flavonoids. TG thermograms of PLA and PLA/*M. officinalis* fibrous mats showed one decomposition peak. The thermal decomposition of electrospun PLA mat started at 355 °C and ended at 423 °C due to the decomposition of the polyester. The presence of *M. officinalis* into the PLA shifts the thermal degradation temperature to lower temperatures. The thermal degradation of electrospun PLA/*M. officinalis* fibrous mat began at 312 °C and ended at 354 °C. The residual weight at 800 °C was 0.12% for the PLA mat and 7.19% for the PLA/*M. officinalis* electrospun material.

### 3.8. Determination of Antioxidant Activity

Oxidative stress plays major role in the pathogenesis of many neurological diseases, including Alzheimer’s disease, Parkinson’s disease and Huntington’s disease; therefore, it has been suggested that antioxidants that could counter cellular oxidative stress within the nervous system could be a potential treatment. Some plant extracts possess strong antioxidant activity [52]. It is known that *Melissa officinalis* is a rich source of antioxidants, in particular from the group of phenolic compounds [53].

A recognized method for evaluating the antioxidant activity of plant extracts is DPPH free radical scavenging [54]. The capacity of the plant extractives to donate hydrogen atoms was assessed using the decolorization of a solution of 2,2-diphenyl-1-picrylhydrazyl. In a methanol or ethanol solution, DPPH creates a violet or purple color that, in the presence of antioxidants, fades to varying colors of yellow. The absorbance of the solutions was measured spectrophotometrically at 517 nm.

**Figure 8:**
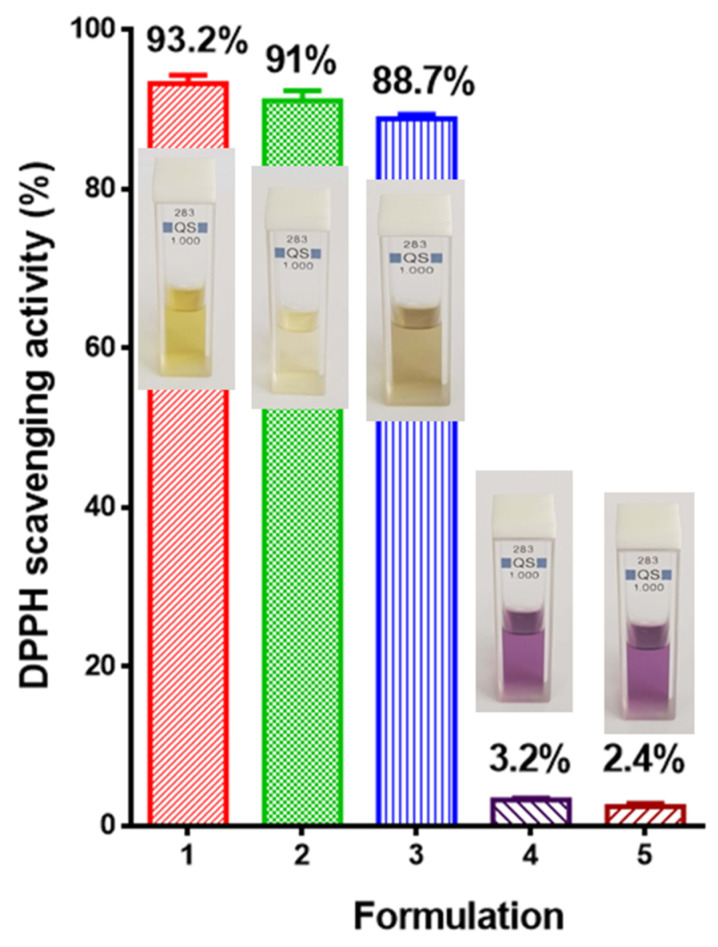
Antioxidant activity of: 1—ethanol solution of *M. officinalis* extract, 2—PLA/PEG/*M. officinalis* mat, 3—PLA/*M. officinalis* mat, 4—PLA/PEG mat and 5—PLA mat. Digital photographs of the corresponding solutions are presented as images as well.

The DPPH scavenging ability of PLA, PLA/PEG, PLA/*M. officinalis* and PLA/PEG/*M. officinalis* mats were determined. The DPPH capacity of the crude extract was assessed as well. As could be seen from Figure 8, the ethanol solution of *M. officinalis* extract showed the highest antioxidant activity (93.2% ± 2.1%). After being in contact with PLA/*M. officinalis* and PLA/PEG/*M. officinalis*, the DPPH solution color changed to yellow and the absorbance of the DPPH radical was dropped by 88.7% ± 1.4% and 91% ± 2.2%, respectively. In contrast, upon contact with PLA and PLA/PEG fibrous materials, the absorbance of the radical decreased by 3.2% ± 0.15% and 2.4% ± 0.2%, respectively, revealing the low antioxidant activity of the polymer materials itself. Moreover, after 30 min of exposure of the PLA and PLA/PEG to the DPPH solution, no significant change in the violet color of the DPPH solution was observed. The results on the antioxidant capacity of the prepared materials revealed that the polymer materials loaded with *M. officinalis* extract possessed high antioxidant activity comparable to that of the crude extract.

## 4. Conclusions

Novel PLA and PLA/PEG-based fibrous materials containing *M. officinalis* plant extract were fabricated by applying the electrospinning method. The average fiber diameters depend on the composition of the spinning solutions and the resulting fibers. It was established that the addition of the extract slightly enhanced the values of the dynamic viscosity, increased the mean fiber diameters and the hydrophobicity of the fibers while the addition of the water soluble polymer–PEG resulted in decrease of the spinning solutions’ viscosity values and imparted hydrophilicity to the fabricated fibrous materials. The PLA/PEG/*M. officinalis* mat and PLA/*M. officinalis* mat displayed strong antioxidant activity comparable to that of the crude extract. The obtained results reveal that the created novel fibrous materials loaded with the *M. officinalis* plant extract are prospective for use in wound dressing applications.

## Figures and Tables

**Table 1:** Phenolic compounds tentatively identified in *M. officinalis* extract by LC-MS in negative ionization mode.

Peak No.	Rt (min)	[M-H]^−^ (*m*/*z*)	Tentative Identification	Ref.
1	3.0	191	Quinic acid	[43]
2	5.1	197	3-(3,4-dihydroxyphenyl)-lactic acid	[44,45]
3	6.5	311	Caftaric acid	[45]
4	12.8	179	Caffeic acid	Standard
5	21.4	473	Chicoric acid	[45]
6	30.4	719	Sagerinic acid	[46]
7	32.2	439	Sulphated rosmarinic acid	[46]
8	34.1	359	Rosmarinic acid	Standard
9	35.5	461	Luteolin 7-O-glucuronide	[44]
10	41.6	537	Lithospermic acid A	[46]
11	45.2	717	Salvianolic acid isomer	[46]
12	52.2	715	Salvianolic acid C derivative	[46]

## Data Availability

Not applicable.

## Supplementary Materials

The following supporting information can be downloaded at: https://www.mdpi.com/article/10.3390/polym15051070/s1, Figure S1: ATR-FTIR spectra of *M. officinalis* (powder), PLA/PEG fibrous mat and PLA/PEG/*M. officinalis* fibrous mat.
