# Supplementary material for: Electrospun PLA-Based Biomaterials Loaded with Melissa officinalis Extract with Strong Antioxidant Activity
Source: Polymers (Basel). 2023 Feb 21;15(5):1070. doi: 10.3390/polym15051070 (PMC10007429; doi:10.3390/polym15051070)
Supplement: Supplementary file 1 [file polymers-15-01070-s001.zip › polymers-2221800-supplementary.pdf]

Supplementary material

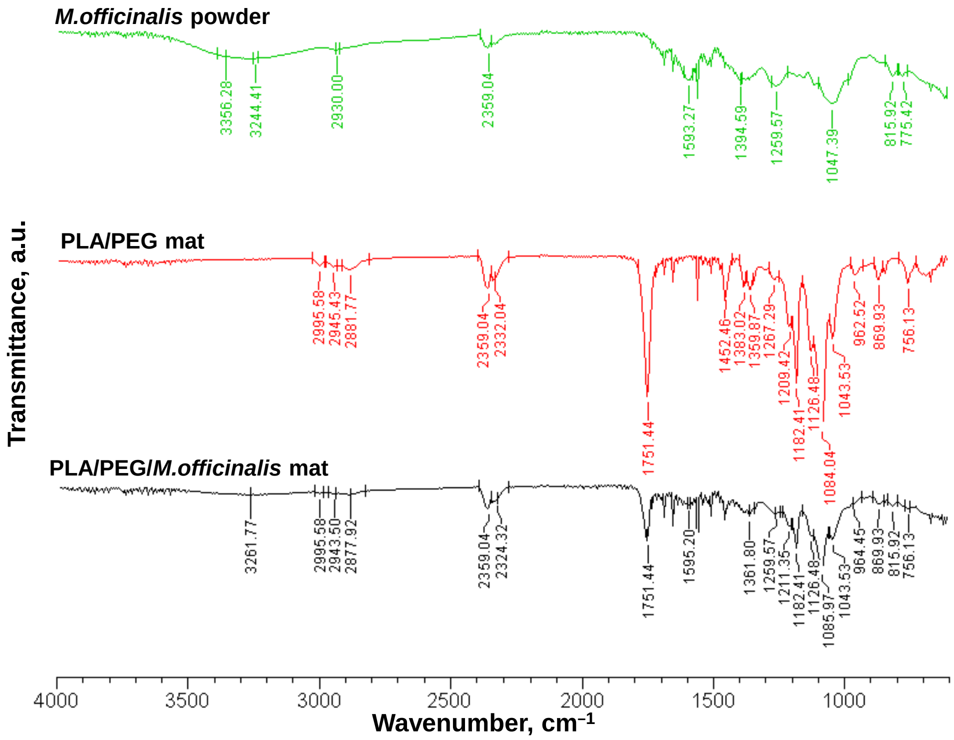

Figure S1. ATR-FTIR spectra of *M. officinalis* (powder), PLA/PEG fibrous mat and PLA/PEG/M. *officinalis* fibrous mat.
